# The application of improved densenet algorithm in accurate image recognition

**DOI:** 10.1038/s41598-024-58421-z

**Published:** 2024-04-15

**Authors:** Yuntao Hou, Zequan Wu, Xiaohua Cai, Tianyu Zhu

**Affiliations:** Heilongjiang Academy of Agricultural Machinery Sciences, Heilongjiang Academy of Agricultural Sciences, Harbin, 150081 China

**Keywords:** Image recognition, Neural network, DenseNet, Data parallelism, Computational science, Computer science, Software

## Abstract

Image recognition technology belongs to an important research field of artificial intelligence. In order to enhance the application value of image recognition technology in the field of computer vision and improve the technical dilemma of image recognition, the research improves the feature reuse method of dense convolutional network. Based on gradient quantization, traditional parallel algorithms have been improved. This improvement allows for independent parameter updates layer by layer, reducing communication time and data volume. The introduction of quantization error reduces the impact of gradient loss on model convergence. The test results show that the improvement strategy designed by the research improves the model parameter efficiency while ensuring the recognition effect. Narrowing the learning rate is conducive to refining the updating granularity of model parameters, and deepening the number of network layers can effectively improve the final recognition accuracy and convergence effect of the model. It is better than the existing state-of-the-art image recognition models, visual geometry group and EfficientNet. The parallel acceleration algorithm, which is improved by the gradient quantization, performs better than the traditional synchronous data parallel algorithm, and the improvement of the acceleration ratio is obvious. Compared with the traditional synchronous data parallel algorithm and stale synchronous parallel algorithm, the optimized parallel acceleration algorithm of the study ensures the image data training speed and solves the bottleneck problem of communication data. The model designed by the research improves the accuracy and training speed of image recognition technology and expands the use of image recognition technology in the field of computer vision.Please confirm the affiliation details of [1] is correct.The relevant detailed information in reference [1] has been confirmed to be correct.

## Introduction

With the explosive growth of digital images, the increasing demand for artificial intelligence, and the popularization of devices such as smartphones, image data has become an important information carrier. Image recognition (IR), also known as image classification, is an important research direction in the field of computer vision^1^. IR is an important tool to promote the automation process in the industry, and the organization and analysis of visual data is more accurate than manual identification and inspection. The automated understanding and analysis of image data helps to realize digital intelligent applications such as automatic driving and intelligent security, which has great social and economic benefits. Currently, IR technology is widely used in industrial, medical, military, and transportation fields, including product quality inspection, medical diagnosis, surgical assistance, target detection and tracking, face and recognition of important scenes. IR technology can improve quality control of manufactured products, diagnosis and analysis of medical imaging data, improve business user experience, and assist in surveillance and monitoring in transportation and power industries^2^. Existing IR technologys are broadly categorized into supervised learning, unsupervised learning, and self-supervised learning, and commonly used technologies include Bayes, decision tree, support vector machine (SVM), and neural network algorithms. Bayes usually performs image classification and matching by calculating the posterior probability of independent features of an image, but the assumption of its principle has a negative impact on the classification effect. Deep learning-based IR technologies usually utilize large-scale deep convolutional neural networks (CNNs) to automatically learn image features, and simplify the complex IR process through multilayer nonlinear processing. However, there are still problems of low recognition efficiency, poor recognition accuracy, sparse feature expression, redundant information, and overly complex classifiers, which limit the effectiveness of its application in accurate IR^3,4^.

To solve these problems, on the one hand, the study proposes an improved strategy for feature reuse in dense convolutional network (DenseNet), which reduces the complexity of the model. On the other hand, in response to the communication bottleneck problem in traditional synchronous data parallel (SDP) algorithms, the parameters of each layer of the traditional algorithm are independently updated. The research employs the gradient quantization (GQ) method to compress communication data and improve the acceleration efficiency of parallel data. This is achieved with the quantization error optimization algorithm.

The innovation of the research is mainly reflected in two aspects. On the one hand, the research proposes an improvement strategy for the traditional DenseNet model to reduce the number of model parameters and simplify the model complexity, which helps to reduce the training cost of the network model. On the other hand, the research changes the parameter updating mode of the parallel algorithm, realizing the overlap of the communication and computation time. And it reduces the size of the communication data with the help of GQ to improve the parallel efficiency of the model in a multifaceted way. The results of this research not only expand the technical means in the field of IR, but also enrich the theoretical research results in the field of DenseNet and parallel computing.

The research content mainly includes five parts. The first part introduces the research background and objectives. The second part is a review of the current research status of IR and classification issues both domestically and internationally. The third part proposes an IR algorithm based on DenseNet, and improves the SDP acceleration training algorithm based on GQ. The fourth part verifies the performance of DenseNet’s IR algorithm. The fifth part summarizes the research experimental results. This model is expected to achieve accurate IR and alleviate the low efficiency in distributed training, thereby improving the running speed of the model.

## Related works

In the information age, IR technology has demonstrated powerful practical functions, and how to establish better IR and classification models has attracted more and more experts’ attention. To promote the accuracy of distinguishing cashmere and wool, Zhu et al. established an IR model based on multi feature selection and random forest. The model utilized a combination of correlation, principal component analysis, and weight coefficients to select important and sensitive features. Finally, the improved random forest algorithm was used to classify images. The research outcomes demonstrated that the IR model made the classification accuracy of cashmere and wool higher, about 90%^5^. Zhu et al. designed an IR method for cashmere and wool fibers based on an improved Xception network. First, the deep features of the fiber image were extracted using the Xception network, and then the improved Swish activation function was used to reduce the over-fitting phenomenon of the entire connection layer. The laboratory findings indicated that the IR accuracy of this network was 98.95%, which was 2% more than the traditional Xception network. The extraction of fiber feature information was more complete, and the IR effect has been improved^6^. To assist fishermen in managing the fishery industry, it needed to promptly eliminate diseased and dead fish, and prevent the transmission of viruses in fish ponds. Okawa et al. designed an abnormal fish IR model based on deep learning, which used fine-tuning to preprocess fish images appropriately. It was proved through simulation experiment that the abnormal fish IR model has improved the recognition accuracy compared to traditional recognition models, and the recall rate has increased by 12.5 percentage points^7^. To improve the recognition efficiency and accuracy of existing IR algorithms, Sun et al. introduced Complete Local Binary Patterns (CLBP) to design image feature descriptors for coal and rock IR. The algorithm used local grayscale median to replace the original CLBP center pixel grayscale, and second-order difference to replace binary difference, simultaneously it utilized deep learning to achieve nonlinear changes in data and deep feature extraction. The experimental results showed that the improved CLBP algorithm raised the recognition accuracy to 94.3%. Recognition efficiency was increased and time consumption was reduced by 71.0%^8^.

To improve the accuracy of stroke imaging diagnosis, Hou et al. designed a deep learning based 3D residual IR model for stroke lesions in medical imaging. The model combined attention mechanisms and residual networks, and utilized 3D convolutional kernels to learn continuous information between image sequences. The experimental results showed that the model could accurately identify whether stroke lesions were contained in medical images, with an average accuracy, sensitivity and specificity of 88.69%, 87.58%, and 90.26%, respectively. The classification performance of IR was significantly better than that of 2D CNNs, and this model had certain practical value^9^. Power line icing seriously endangered the operation safety of power system. To accurately and efficiently identify power grid images, Hao et al. proposed a weak supervision and phased transfer learning method, which fused multi-dimensional features to reduce the interference of background and camera occlusion. The experimental results showed that this method could identify different types of line covers, with recognition accuracy and recall rates of 86.6% and 91.3%, respectively, and a recognition speed of 8 ms per amplitude^10^. To improve the face IR technology, Rangayya et al. fused the SVM and the improved random forest to design a face IR model. The model utilized active contour segmentation and neural networks to segment facial images. The experiment findings denoted that the model had better recognition performance than existing common facial recognition algorithms. The accuracy, precision, recall, and F1 value indicators reached 99.2%, 96%, 98%, and 96%, respectively^11^. Gao proposed an IR method that combined deep learning algorithms with SVM. The method could achieve multi-level classification and deep extraction of image features. After preprocessing operations such as color component compensation, image denoising, and threshold segmentation, the extracted features were compared with standard features to gain the final IR result. The research outcomes expressed that the recognition rate of this method had been improved by 6.6%^12^. Wang et al. compared the IR effects of SVMs and CNNs for machine learning, respectively, and found that the accuracy of SVM was 0.88 and that of CNNs was 0.98 on the large-scale dataset. On the small-scale dataset COREL1000, the accuracy of SVM was 0.86 and that of CNNs accuracy was 0.83. The two models had different adaptations for different size datasets^13^. Sarwinda et al. designed a residual network-based IR model for the detection of colon cancer. Residual network-18 and Residual network-50 were trained on the colon gland image dataset to differentiate the benign and malignant colon tumours, respectively. The experimental results showed that Residual network-50 performed more reliably in terms of accuracy, sensitivity and specificity values^14^. Jacob and Darney designed a CNN-based IR model to improve the accuracy of IR in IoT, and evaluated the experiments on the IoT image dataset practical appropriateness in IoT systems^15^.

Some experts have conducted training and learning for general tasks, and Kommaraju et al. have explored unsupervised representation learning methods for biomedical texts, introducing new pre-training tasks from unlabeled data. The experimental results showed that the pre-training task proposed in the study could significantly improve the model performance and reduce the train-test mismatch between the pre-training task and the downstream quality assurance task^16^. To achieve large-scale meta-learning, Bansal et al. designed multiple self-supervised task distributions. The experimental results showed that the variety, difficulty, type, field and curriculum of tasks could change task assignment meaningfully^17^. Bansal et al. investigated the application of residual-dense network architecture in single image super resolution, and conducted performance tests on the residual-dense network architecture by studying different blocks of the network architecture and analyzing various loss indicators. The research results showed that the architecture was effective compared with the existing advanced models^18^. In addition, Gunasekaran and Jaiman also studied the problem of image classification under occlusion objects. Taking autonomous vehicles as the research object, they used existing advanced IR models to test the robustness of different models on occlusion image dataset^19^. In addition, the research was also carried out on the classification task of news texts. A pre-trained text classification model was used to classify more than 350,000 news articles according to the publication time, and the experimental results showed that the performance of this model was superior to other basic models^20^. Gunasekaran et al. carried out relevant research on the configuration of the management database system and designed a supervised and unsupervised machine learning method for automatic problem solving to complete the generation of configuration. The model simplified the selection of indicators and improved the accuracy of the algorithm^21^.

In summary, various fields have extensively studied models for IR classification and processing, resulting in improved recognition accuracy. However, due to the massive scale of IR projects and the distribution of images, actual image datasets face an imbalance problem. As a result, the model still exhibits various overfitting phenomena during the training process. Faced with massive image data, the huge computational workload and long training time still leave significant room for improvement in the timeliness of the model. Improvement of recognition accuracy should also focus on the improvement of recognition efficiency, and should not satisfy the accuracy improvement and consume huge computational cost. In this regard, the study was carried out for the change optimization of the feature extraction module of DenseNet, and at the same time, the image processing adaptability of the parallel algorithm was improved.

## Design of an accurate IR model combining densenet and GQ

IR technology has been applied to many complex application scenarios, and the requirements for IR algorithm models are also increasing. How to extract effective features from image information while minimizing training costs has become a research focus in the image development. On the one hand, the research improve the feature reuse method of DenseNet, and on the other hand, it also improves the parallel training mode of synchronous data by using the GQ to compress the communication data and accelerate the algorithm training speed.

### Design of IR and classification algorithm based on improved densenet

The neural network model can automatically learn the target features, has the ability to directly process high-dimensional data as well as adaptive adjustment of parameters, excellent feature extraction ability, generalization ability, data processing ability to meet the requirements of the IR technology. Therefore, the research adopts the deep neural network model as the basis for constructing the IR model.

**Figure 1:**
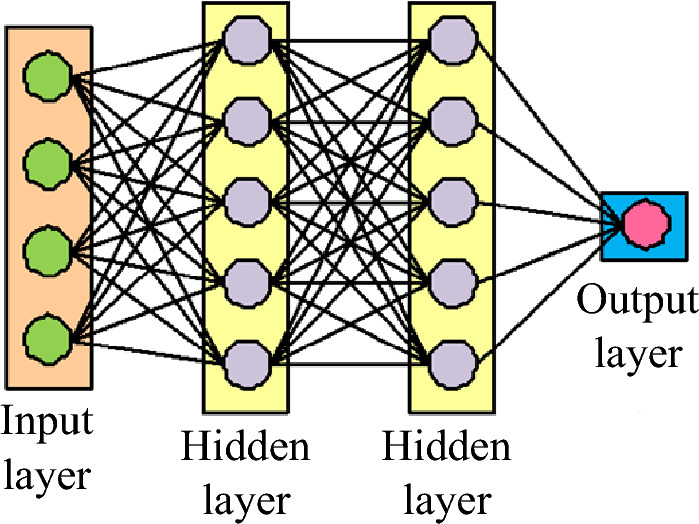
Schematic diagram of CNN structure.

Deep learning is composed of multiple layers of neurons and is a deepening of neural network models, with more network layers and model fitting capabilities. CNNs, as a typical deep learning method, are widely used in IR and classification. Google Perception Net, Visual Geometry Group (VGG), Residual Neural Network, and DenseNet are all developed from CNNs^22–24^. CNN is a deeply supervised machine learning model with strong adaptability, especially good at mining local features of data, extracting global features and carrying out classification processing (Fig. 1). After a number of operations, the feature map is finally obtained. At the early stage of the training of the CNN, the feature map will be extracted to the edge and texture information of the image. As the network goes deeper, the information with more semantic information level will be extracted, and after several feature extraction operations, the CNN will complete the recognition task of the whole image.

**Figure 2:**
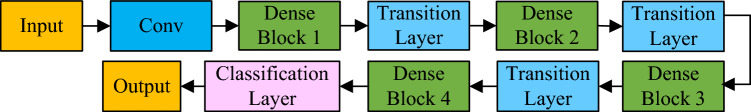
DenseNet structure diagram.

DenseNet is a deep learning neural network architecture where each layer is connected to all previous layers and information can flow fully and efficiently through the network. This feature allows the DenseNet network to better learn and understand the details and structure of an image. Due to the dense connectivity, the DenseNet network enables feature reuse, which improves the algorithm’s feature representation and learning efficiency. In addition, the DenseNet network structure is simple and has a small number of parameters, which solves the problem of gradient vanishing that commonly exists in neural network models. Therefore, the study selected DenseNet as the object of research, and the DenseNet structure is shown in Fig. 2^25,26^.

The training of CNNs usually uses gradient values to update parameters from the back to the front. According to the chain rule, if the product of the neuron weights in each layer and the residuals passed from the previous layer of the network is less than 1, as the amount of model layers increases, the gradient value will gradually approach 0 during the transmission process. It is the phenomenon of gradient disappearance, also known as gradient dispersion. The use of the Corrected Linear Unit activation function in the CNN can reduce the gradient disappearance, and the residual module can also be used. The model is not learning its own identity mapping. The DenseNet draw inspiration from this idea by adding quick connections in the network model, where gradient values are transmitted through quick connections in the network. At the same time, the DenseNet also uses feature reuse to reduce the amount of model parameters^27–29^.

The output expression of a layer of the DenseNet is shown in Eq. (1), where $x_{0}$ means the input of the neural network. $H_{l}$ stands for nonlinear transformation, including convolution, activation function, etc. $x_{l}$ means the output of the network $l$ layer.

1 $$x_{l} = H_{l} \left( {x_{0} ,x_{1} ,x_{2} ,...,x_{l - 1} } \right)$$

**Figure 3:**
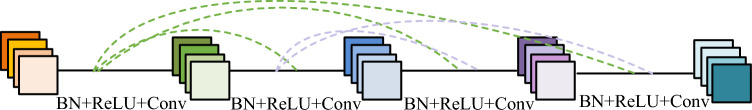
Schematic diagram of dense connection module.

The DenseNet includes four parts: dense connection module, bottleneck layer, conversion layer, and classifier. The feature map undergoes a dimensionality reduction operation through the pooling layer of the CNN, and the size of the feature map is reduced by half. The DenseNet concatenates the feature map through a dense connection module, and the feature map is dimensionally reduced by the conversion layer of the dense connection module which is shown in Fig. 3^30^.

The number of characteristic maps of $l$ layer is shown in Eq. (2), where $k$ is a hyperparameter, representing the number of characteristic maps of bottleneck layer, that is, growth rate. $k_{0}$ expresses the number of input feature maps.

2 $$k_{l} = k_{0} + k\left( {l - 1} \right)$$

**Figure 4:**
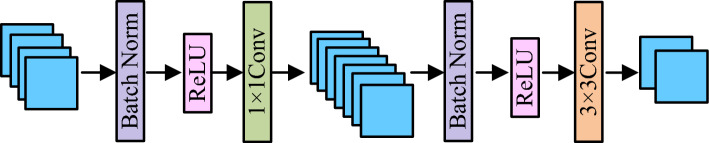
Schematic diagram of bottleneck layer structure.

The DenseNet bottleneck layer consists of two convolutional layers, $1 \times 1$ and $3 \times 3$, while $1 \times 1$ convolution shortens the model width. Before the convolution operation, the feature image is normalized and preprocessed, and the activation function is used for nonlinear transformation. The bottleneck layer is shown in Fig. 4. Similarly, the conversion layer also incorporates $1 \times 1$ convolution, reducing the number of model feature maps determined by the compression coefficient. It can be freely adjusted according to model settings.

Although the DenseNet network model largely cuts down the number of parameters and overcomes the gradient vanishing, there are still some shortcomings in the DenseNet. Firstly, the reuse of low-level features extracted by the DenseNet will result in a decrease in model parameter efficiency. Secondly, the DenseNet network contains a lot of feature map concatenation operations, which ultimately leads to excessive memory usage and insufficient storage space, which further affects the efficiency of model training. In this regard, research has optimized the DenseNet network, with two improvement ideas. Firstly, it is to reduce the scale of the DenseNet network and a portion of the feature map. The second is to reduce the number of reused feature maps during feature reuse, and to study using a random method to select randomly discarded feature maps.

Firstly, it adjusts the growth rate, and the improved input feature map quantity expression is shown in Eq. (3). Where, $\alpha_{i}$ denotes the ratio parameter of the $i$ th layer’s feature maps entering the $l$ layer, and $k_{0}$ indicates the amount of feature maps input to the dense connection module.

3 $$k_{l} = \alpha_{0} k_{0} + k\sum\limits_{i = 1}^{l - 1} {\alpha_{i} }$$

The amount of feature maps reduced in the $i$ th layer is shown in Eq. (4), and the size of $\alpha_{i}$ determines the number of feature maps input to the $l$ layer.

4 $$k_{d} = k\left( {1 - \alpha_{i} } \right), \, \alpha_{i} = \left\{ {\begin{array}{*{20}c} { - 0.05\left( {l - i} \right) + 1.05 \, i \ge l - 10} \\ {0.5 \, i < l - 10} \\ \end{array} } \right.$$

After adjustment, from Eq. (4), as the interval between layers $i$ and $l$ increases, $\alpha_{i}$ will gradually decrease. But when the distance between the two exceeds 10 layers, $\alpha_{i}$ is set at a constant value of 0.5, and $\alpha_{i}$ is not too small, still ensuring the extraction of low-level features from the previous network layer.

Then it adjusts the network width of the DenseNet. The network width and depth of the DenseNet determine the parameter quantity of the DenseNet, with the deeper and wider the depth, the more parameters DenseNet has. The study adjusts the growth mode of DenseNet by adjusting the way the width of the DenseNet changes with depth. After improvement, the compression coefficient of the conversion layer in the DenseNet is set to 1, and the growth mode is changed to a gradually widened network growth mode.

### Design of parallel accelerated training algorithm for synchronous data based on GQ

The learning and training of deep neural networks usually involves a large number of parameters, and the training is computationally intensive and time-consuming. However, the processing task of deep learning continues to escalate. To meet the needs of increasingly complex application scenarios, the size of deep learning network models has increased further and the training process has become more complex. In a nutshell, the development of the Internet and big data technologies has led to an extremely rapid expansion of the datasets available for model training, as well as an increase in the size of the models. In order to speed up the training of network models and save training costs, large-scale computing clusters and parallel computing are often used to further accelerate training. Distributed training solves this development contradiction by dividing the data set into multiple parts and then training the model in parallel on multiple computing nodes. This approach can greatly reduce training time, improve the accuracy and reliability of the model, and utilize the computing resources of multiple computing nodes to process large-scale datasets. Model parallelism is where the distributed nodes store different parameters with the same input layer. Data parallelism is the exact opposite, where each distributed node stores the same parameters with different data in the input layer. The study was developed based on the data parallelism accelerated training method to speed up the training of neural network models and reduce communication costs as much as possible^31,32^.

**Figure 5:**
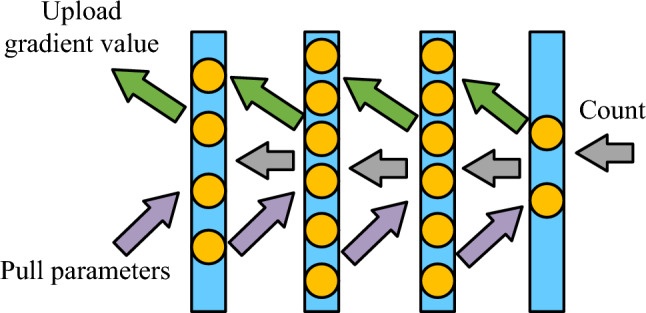
Overlapping computation and communication time iteration.

The main factors affecting the communication time of the model include the amount of communication data and network bandwidth, and a number of communication data will increase with the increase of network model parameters. However, the network bandwidth provided by general Ethernet cannot directly support linear acceleration. In response to these two causes of communication bottlenecks, research has improved the SDP algorithm. The improved calculation and communication is shown in Fig. 5.

**Figure 6:**
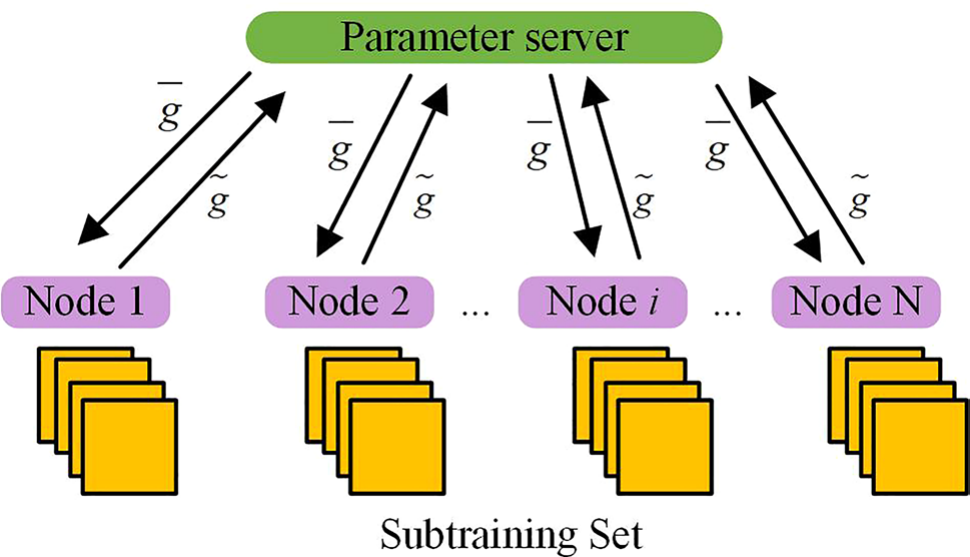
Schematic diagram of data parallel algorithm based on GQ.

As seen in Fig. 5, the parallel algorithm in the neural network adds a communication phase: uploading gradient values and pulling parameter stages. The study independently updates the parameters of each layer in traditional SDP algorithms. When the gradient value of a network layer parameter is calculated, there is no need to wait for the calculation results of other layers. Instead, the gradient value is immediately uploaded to the parameter server for parameter updates, achieving parallel computation and communication processes. Regarding the reduction way of communication data, the GQ method is adopted in the study, and the data parallel algorithm based on the GQ is shown in Fig. 6. Because the number of gradient values used in communication cannot be changed, the purpose of the GQ method is to reduce the accuracy of gradient values^33–35^.

The quantitative methodology used is expressed in Eq. (5), where $g$ represents the gradient vector. $\widetilde{g}$ means the quantization gradient value of $g$. $\lambda$ expresses the minimum gradient value of $g$. $d$ stands for the length of a single quantization interval.

5 $$\widetilde{g} = Q \cdot d + \lambda$$

The definition of minimum gradient value and quantization interval is shown in Eq. (6), where $\min \left( g \right)$ and $\max \left( g \right)$ represent the minimum and maximum gradient values of $g$, respectively. $s$ refers to an adjustable positive integer, representing the segmentation interval of the gradient vector, which determines the compression effect of communication data.

6 $$\left\{ {\begin{array}{*{20}c} {\lambda = \min \left( g \right)} \\ {d = \frac{\max \left( g \right) - \lambda }{s}} \\ \end{array} } \right.$$

The expression of the GQ value corresponding to the number $Q$ is shown in Eq. (7). Where $l \in \left\{ {0,1,...,s} \right\}$ and $p$ indicate the probability of using quantization values to approximate the gradient value.

7 $$Q = \left\{ {\begin{array}{*{20}c} {l \, 1 - p} \\ {l + 1 \, p = \frac{g - \lambda }{d} - l} \\ \end{array} } \right.$$

The mathematical expectation for the numbered $Q$ and GQ values is shown in Eq. (8). Because the research reduces the accuracy of gradient values, there may be errors in parameter updates. However, according to Eq. (8), the mean of $g$ and $\widetilde{g}$ is the same. Therefore, this error deviation is compensated for during the model iteration.

8 $$\left\{ \begin{gathered} E\left[ {\widetilde{g}} \right] = E\left[ {Q \cdot d + \lambda } \right] = E\left[ Q \right] \cdot d + \lambda = g \hfill \\ E\left[ Q \right] = \frac{g - \lambda }{d} \hfill \\ \end{gathered} \right.$$

After receiving the gradient vectors uploaded by all network layers, the parameter server calculates the average of the gradient values, as shown in Eq. (9). Where $N$ represents the number of calculation nodes, $\overline{\lambda }$ denotes the average of the minimum values of each gradient vector, and $\overline{g}$ indicates the average value of the quantization gradient.

9 $$\overline{g} = \frac{1}{N}\sum\limits_{i = 1}^{N} {\widetilde{g}_{i} } = \frac{1}{N}\sum\limits_{i = 1}^{N} {\left( {Q_{i} \cdot d_{i} + \lambda_{i} } \right) = \frac{1}{N}\sum\limits_{i = 1}^{N} {\left( {Q_{i} \cdot d_{i} } \right) + \overline{\lambda } } }$$

Equation (9) is simplified by replacing the quantization interval of nodes with the average value of the quantization interval. The approximate expression is shown in Eq. (10), where $\overline{Q}$ means the average value of the quantization number.

10 $$\overline{g} = \overline{d} \cdot \frac{1}{N}\sum\limits_{i = 1}^{N} {Q_{i} } + \overline{\lambda } = \overline{d} \cdot \overline{Q} + \overline{\lambda }$$

Finally, the parameter server uses $\overline{g}$ to update the model parameters, as shown in Eq. (11). Where $t$ represents the number of iterations, and $\eta$ is the learning rate. In addition, the downlink communication cannot compress the communication data volume by reducing gradient accuracy.

11 $$w^{t + 1} = w^{t} - \eta \overline{g}^{t}$$

The decrease in gradient accuracy will cause quantization error, as shown in Eq. (12), where $i$ means the computing node.

12 $$r_{i} = g_{i} - \widetilde{g}_{i}$$

It uses quantization error to correct the parameter update, and sums the quantization error with the average quantization gradient to obtain the corrected gradient value. The update process is shown in Eq. (13), and $t$ indicates the number of model iterations.

13 $$w_{i}^{t + 1} = w_{i}^{t} - \eta \left( {\overline{g}^{t} + r_{i}^{t} } \right)$$

To more intuitively analyze the impact of quantization errors on model parameter updates and model performance, a derivation analysis is conducted based on traditional synchronous data algorithms. After adding quantization parameters, the parallel average gradient calculation of traditional synchronous data is shown in Eq. (14). Where $g_{i}^{*}$ expresses the original gradient of the node, $\overline{r}$ denotes the mean quantization error, and $\overline{{g^{*} }}$ represents the average gradient of traditional synchronous data algorithms^36–38^.

14 $$\overline{{g^{*} }} = \frac{1}{N}\sum\limits_{i = 1}^{N} {g_{i}^{*} } = \frac{1}{N}\sum\limits_{i = 1}^{N} {\left( {\widetilde{{g_{i} }} + r_{i} } \right)} = \overline{g} + \overline{r}$$

The parameter updates of traditional synchronous data algorithms are shown in Eq. (15), where $w^{*}$ refers to the node model parameters of traditional synchronous data algorithms.

15 $$w^{*t + 1} = w^{*t} - \eta \left( {\overline{g}^{t} + \overline{r}^{t} } \right)$$

According to Eq. (15), the calculation of the average parameter value of the model nodes can be seen in Eq. (16).

16 $$\frac{1}{N}\sum\limits_{i = 1}^{N} {w_{i}^{t + 1} } = \frac{1}{N}\sum\limits_{i = 1}^{N} {\left[ {w_{i}^{t} - \eta \left( {\overline{g}^{t} + r_{i}^{t} } \right)} \right]} = \frac{1}{N}\sum\limits_{i = 1}^{N} {w_{i}^{t} - \eta \overline{g}^{t} - \eta \overline{r}^{t} }$$

Due to the initialization of model parameters, i.e. $\frac{1}{N}\sum\limits_{i = 1}^{N} {w_{i}^{0} } = w^{*0}$, before the start of model training. Therefore, the average value of the parameters after the first iteration is shown in Eq. (17), indicating that the average value is the same as the parameter values of traditional synchronous data algorithms.

17 $$\frac{1}{N}\sum\limits_{i = 1}^{N} {w_{i}^{1} } = \frac{1}{N}\sum\limits_{i = 1}^{N} {w_{i}^{0} - \eta \overline{g}^{0} - \eta \overline{r}^{0} } = w^{*1}$$

From this, during the iterative of the algorithm, the parameter values have changed after the quantization error correction, but the average value of the node parameters is always equal to the parameter values in traditional synchronous data algorithms. The parameter value $w_{i}$ of each node is close to the original parameter $w^{*}$, compensating for the loss caused by the decrease in gradient accuracy.

**Figure 7:**
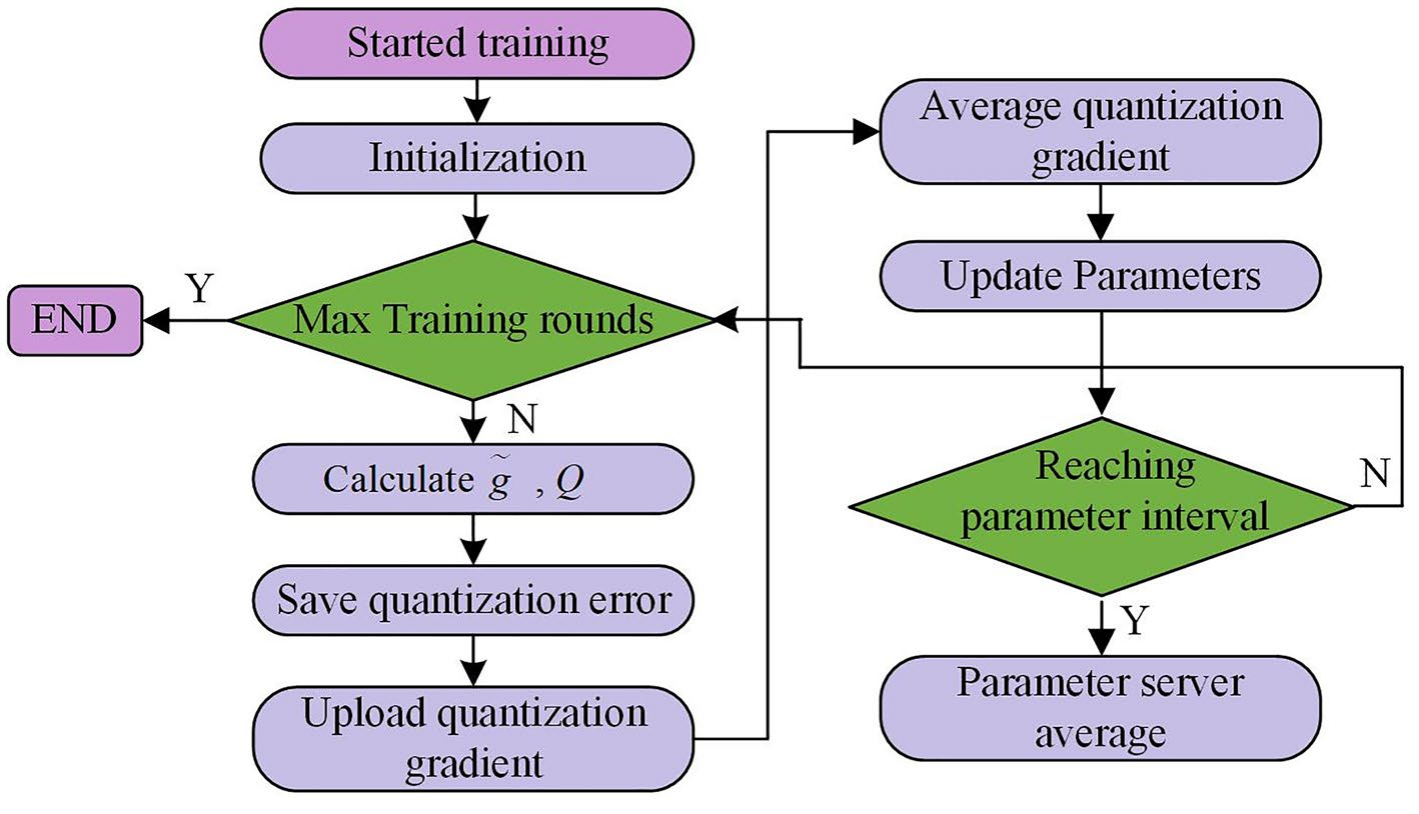
Flow chart of improved SDP algorithm.

Finally, the study adds a parameter average interval $a$ to the SDP algorithm. After $a$ iterations, the parameter server averages the updated parameter values, and the mean returns to the nodes. Each node starts new training from the same mean starting point. The entire improved SDP algorithm flowchart is shown in Fig. 7.

## Improved densenet IR algorithm performance testing

To verify the effectiveness of the IR model designed in this study, a testing experiment was designed to find the influence of network depth on recognition performance. On the other hand, it evaluated and optimized the recognition accuracy and efficiency of research optimization algorithms. The experiments were designed based on the effect of three different depths on the classification effect based on the improved model, including a shallow model with shallow depth and small number of parameters and a large deep model.

### Design of test experiment plan and model parameter setting

The first step is the design of the test programme and the presentation of the model parameters. The three different depths of DenseNet CNNs designed for the study were respectively named DenseNet-50, DenseNet-100, and DenseNet-200. DenseNet-50 included three dense modules, with each dense connection module set with 6 bottleneck layers, a growth rate of 12, and a compression coefficient of 0.5. The fully connected layer used the Softmax function to output prediction probability, and the total number of model parameters was 0.180 M. DenseNet-100 included 100 convolutional layers, with other parameter settings unchanged, but the dense connection module was set with 8, 16, and 24 bottleneck layers, with a total model parameter of 0.540 M. DenseNet-200 included 4 dense connection modules, with 8, 16, 24, and 32 bottleneck layers set, and a growth rate of 24. Other parameter settings remained unchanged. The feature maps output under the parameter settings of the three network models are shown in Table 1, where the third layer of DenseNet-200 is converted into an output feature map of 4 × 4 × 356, 2 × 2 × 356. Dense Block4 output feature map is 2 × 2 × 680.

**Table 1:** Details of model output feature map.

Operation	Conv	Dense block1	Transition layer 1	Dense block2	Transition layer 2	Dense block 3	Classifier
DenseNet-50	16 × 16 × 24	16 × 16 × 81	16 × 16 × 41 8 × 8 × 41	8 × 8 × 92	8 × 8 × 46 4 × 4 × 46	4 × 4 × 98	1 × 1 × 98 1 × 10
DenseNet-100	16 × 16 × 24	16 × 16 × 97	16 × 16 × 49 8 × 8 × 49	8 × 8 × 158	8 × 8 × 79 4 × 4 × 79	4 × 4 × 221	1 × 1 × 221 1 × 10
DenseNet-200	16 × 16 × 48	16 × 16 × 162	16 × 16 × 81 8 × 8 × 81	8 × 8 × 255	8 × 8 × 128 4 × 4 × 128	4 × 4 × 712	1 × 1 × 680 1 × 10

### Analysis of improved densenet IR performance

**Figure 8:**
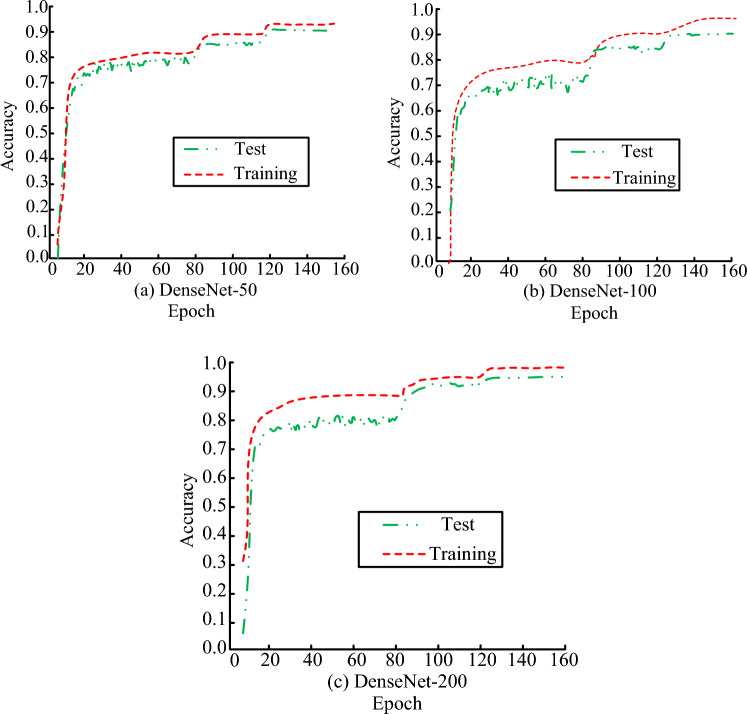
Recognition accuracy of densenet networks with different depths.

The recognition effectiveness of the DenseNet model designed for the study is validated, and the experimental parameter settings and datasets are described below:160 training sessions were conducted on the CIFAR-10 dataset, with a batch size of 64 and an initial learning rate of 0.1. The learning rate was reduced during the 80th and 120th training sessions. Normalization preprocessing and image enhancement operations were operated on images before training. After the training was completed, network testing experiments were conducted on the test set. The accuracy results of the three deep experiments of the DenseNet model are shown in Fig. 8. To ensure completion of testing in each epoch, the study separated the training set from the test set. After updating the model parameters in each epoch of the training set, the weights of the model or the entire model were saved. Then, the performance of the model was evaluated on the test set using the testing function provided in the computational framework. Where, due to the reduced learning rate during 80 training sessions and the reduced number of model parameters during iteration, there was a sharp increase in the accuracy curve of the three models at this point, and the trend of the accuracy change curve of the three models was roughly the same. At the beginning of model training, the network quickly learned the training data and converged to the level near the optimal accuracy curve. As the model continued to train, the accuracy curve eventually stabilized.

When the learning rate was high, the accuracy curve fluctuated greatly. After reducing the learning rate, the curve fluctuation decreased, and the parameter update granularity of the network model decreased, ultimately achieving the optimal accuracy value. The maximum accuracy values of the DenseNet-50, DenseNet-100, and DenseNet-200 were 92.3%, 95.4%, and 97.2%, respectively. As the number of network layers deepened, the accuracy values of the DenseNet-200 increased by 5.31% and 1.88%, respectively, indicating that the deepening of network layers could effectively improve the final recognition performance of the model.

**Figure 9:**
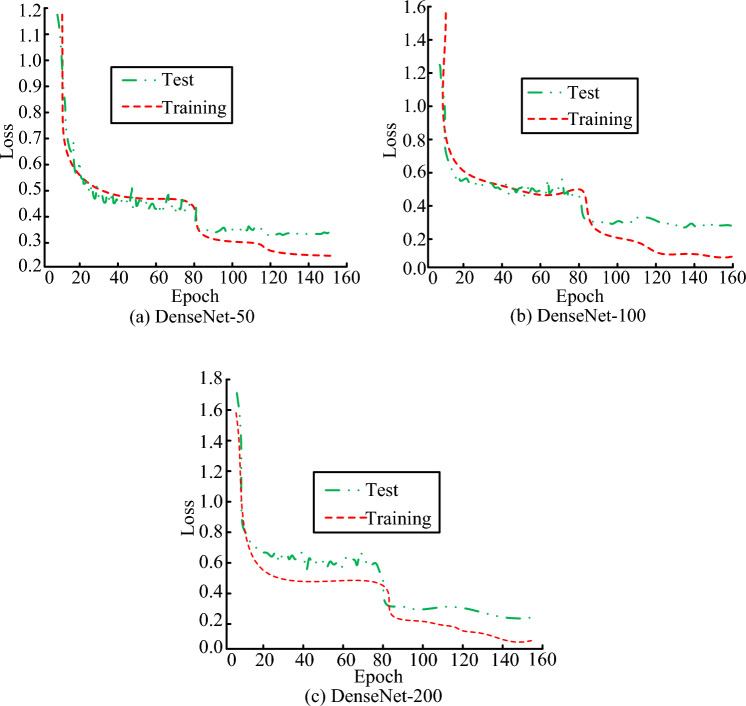
Loss curves of densenet at different depths.

The loss curves of the three networks are shown in Fig. 9. Where the loss curve trend of the DenseNet networks with three different depths was generally consistent. Reducing the learning rate during the 80th training also led to a sharp decrease in the loss rate curve and a decrease in the loss value. The Loss value represented the difference between the predicted and the actual values as the number of training increased. As the amount of training sessions increased, the network’s ability to fit the training data increased. Therefore, the loss curve showed a downward trend and eventually stabilized. The deepening of the network layers could effectively improve the convergence effect of the model.

**Figure 10:**
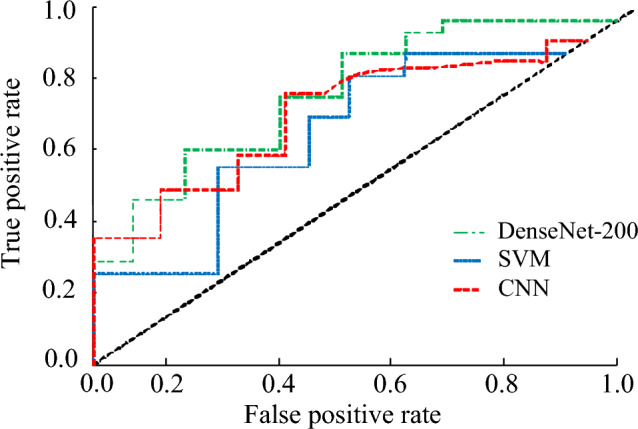
Subject characteristics curves for different models.

The Receiver Operating Characteristic Curve (ROC) is the ratio of the recall rate to the false positive rate, while the Area Under Curve (AUC) value is the area enclosed by the ROC curve and the coordinate axis. The AUC values of the DenseNet-200, SVM, and CNN are shown in Fig. 10. The ROC curve of the DenseNet-200 model was at the top, with an AUC value of 0.945. The AUC values of the SVM and CNN models were 0.821 and 0.803, respectively, which were significantly lower than those of the DenseNet-200 model. In summary, the DenseNet model constructed in the study had good performance in all aspects, and the comprehensive evaluation index performed well for accurate recognition of images.

### Analysis of recognition accuracy and computational efficiency

**Figure 11:**
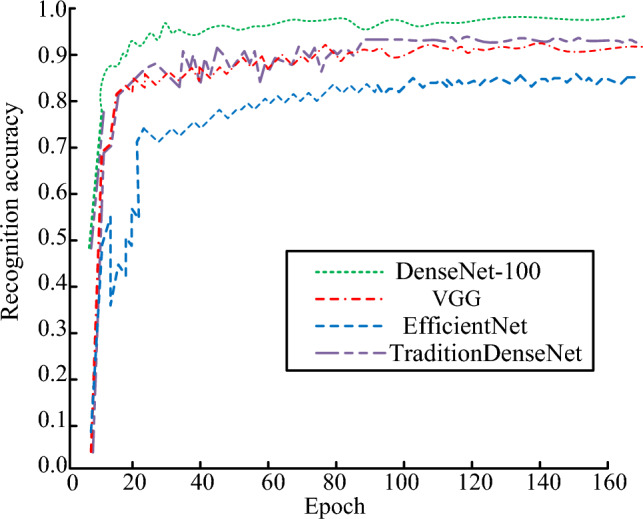
Recognition accuracy of different algorithms.

The DenseNet-100 network was selected to compare the IR accuracy with the CNN VGG and the image classification model EfficientNet, and the dataset was selected from the indoor scene detection dataset MIT-67, the large-scale scene understanding dataset SUN-397, and the medical image dataset. The average recognition accuracies of three kinds of datasets are shown in Fig. 11. As seen in Fig. 11, compared with the traditional DenseNet recognition accuracy, the accuracy of the DenseNet-100 was relatively better, and finally stabilized at 0.986. The recognition accuracies of the other two models were lower than that of the DenseNet-100 on different datasets, with the average recognition accuracy of VGG being 0.924, and that of EfficientNet being 0.849. The DenseNet-100 model showed excellent robust performance.

Performance testing was conducted on the improved SDP algorithm. Compared with traditional SDP algorithms and Stale Synchronous Parallel (SSP) algorithms, the number of nodes was calculated to be 3, where the acceleration ratio referred to the ratio of training speed to a single node. From Table 2, the acceleration ratio of DenseNet-50 improved by using the GQ was relatively small compared to the other two acceleration algorithms, due to the excessive time consumption in the GQ and encoding operations when the network parameters were small. When the network deepened and the number of parameters increased, the parallel acceleration algorithm improved by the GQ performed better in acceleration ratio, far higher than the other two algorithms, with a ratio of 2.81 and 2.61, and a maximum improvement of 1.92. The low acceleration ratio indicated that the algorithm has caused waste of node computing resources. When the acceleration ratio decreased to 0.57 and 0.69, the communication bottleneck was reached.

**Table 2:** Accelerated training time of DenseNet models with different depths.

Model	Training algorithm	Time(s)	Speed up
DenseNet-50	SDP	21.3	2.67
	SSP	21.6	2.63
	GQ-SDP	21.4	2.59
DenseNet-100	SDP	56.2	2.03
	SSP	51.7	1.68
	GQ-SDP	39.5	2.81
DenseNet-200	SDP	397.5	0.57
	SSP	426.3	0.69
	GQ-SDP	84.7	2.61

**Figure 12:**
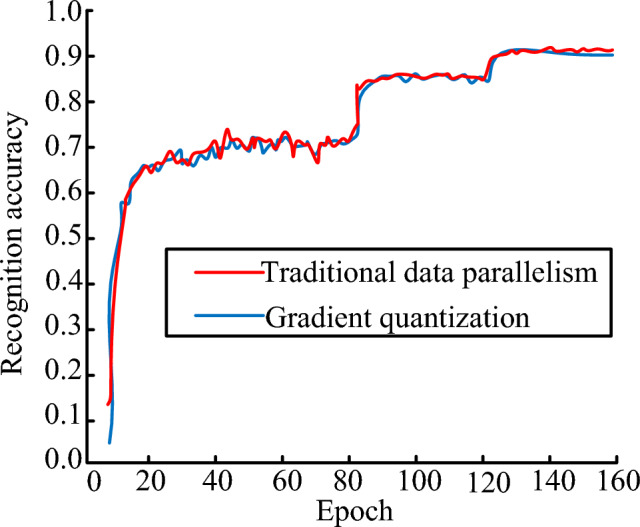
Test accuracy change curve.

In terms of training time, as the network deepened, the difference in training time became increasingly significant. The DenseNet network improved by GQ performed well in computational time. The DenseNet-100 model reduced the computational time by 16.7 s and 12.2 s compared to the other two algorithms, respectively. The DenseNet-200 model took 312.8 s and 341.6 s less time than the other two algorithms, respectively. Finally, the DenseNet-100 was selected to compare the recognition accuracy of traditional data parallel algorithms and GQ improved algorithms. The experimental results were displayed in Fig. 12, where the recognition accuracy curves of the two data parallel processing algorithms were relatively consistent. The curve trends were basically coincident, indicating that different data parallel processing methods did not affect the final IR results.

**Figure 13:**
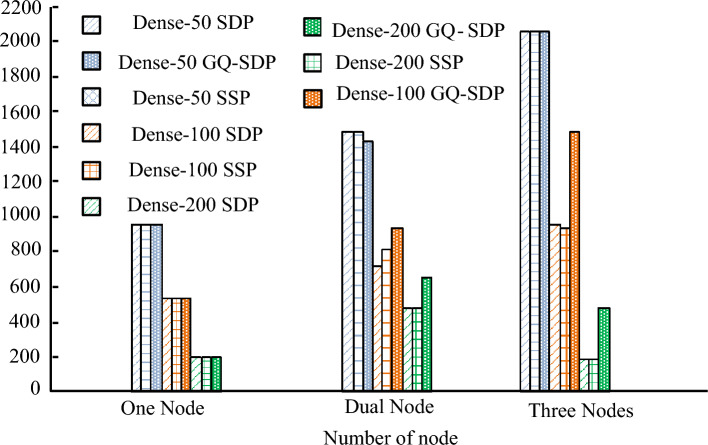
Number of images processed per second by different models.

The results of processing image data per second for different model nodes are shown in Fig. 13. The DenseNet-50 processed the highest number of images, but for different numbers of nodes, the improved GQ-based data parallelism algorithm did not show a greater advantage, with fewer network layers and smaller data sizes. The study’s improved GQ-based data parallelism algorithm did not show a greater advantage for different numbers of nodes, with fewer network layers and smaller data sizes, and failed to reflect the advantages of the study’s constructed model. The DenseNet-200 algorithm gradually decreased the number of images processed at a node count of 3. This indicated that the algorithm suffered from a more serious communication bottleneck, but the GQ improvement method was still able to significantly speed up image processing.

## Conclusion

Aiming at the research dilemma of IR technology, the research designed an accurate and fast recognition model for images based on the DenseNet model, and improved the feature multiplexing of DenseNet as well as the communication data processing of synchronized data parallel algorithm. The performance test experimental results showed that the improved strategy designed by the research improved the efficiency of the model parameters while guaranteeing the recognition effect. The stable values of the accuracy of the DenseNet-50, 100 and 200 were 92.3%, 95.4% and 97.2%, respectively, and the deepening of the number of layers of the network could effectively improve the final recognition effect and convergence effect of the model. The average recognition accuracy of the DenseNet-100 was better than that of VGG and Efficient Net IR models on different datasets. The parallel acceleration algorithm improved by GQ performed better in terms of acceleration ratio, which was much higher than the other two algorithms, with a maximum increase of 1.92. Compared with the traditional SDP algorithm and SSP parallel algorithm, the optimized parallel acceleration algorithm of the study consumed significantly less time, which ensures the training speed of the image data and at the same time solves the bottleneck problem of the communication data. However, the algorithm designed in the study is based on a centralized parameter server architecture. It is necessary to use a more complex parameter server architecture in future research to further improve the algorithm training speed.Author contribution is mandatory for publication in this journal. Please provide the statement.In order to improve the accuracy of image recognition, the study chooses dense convolutional network as the model base framework. On the one hand, in order to reduce the model training cost, a feature reuse improvement strategy is proposed to reduce the number of model parameters and simplify the model complexity. On the other hand, the parameter updating method of the parallel algorithm is changed in order to reduce the communication time; and the size of the communication data is reduced with the help of gradient quantization, which further enhances the efficiency of parallel computation collaboratively. The study enriches the research theory of dense convolutional networks and parallel computing, and improves the application level of image recognition technology.

## Data Availability

The data used to support the findings of this study are available from the corresponding author upon request.
